# Increased serum anti-N-methyl-D-aspartate receptor antibody immunofluorescence in psychiatric patients with past catatonia

**DOI:** 10.1371/journal.pone.0187156

**Published:** 2017-10-26

**Authors:** Chin-Chuen Lin, Yi-Yung Hung, Meng-Chang Tsai, Tiao-Lai Huang

**Affiliations:** Department of Psychiatry, Kaohsiung Chang Gung Memorial Hospital and Chang Gung University College of Medicine, Kaohsiung, Taiwan; Chiba Daigaku, JAPAN

## Abstract

**Objective:**

Anti-N-methyl-D-aspartate receptor (NMDAR) antibody was thought to be the cause of anti-NMDAR encephalitis, with manifestations similar to catatonia and schizophrenia. Anti-NMDAR antibody in neuropsychiatric patients who had catatonia before were investigated in a follow-up evaluation. The intensity of antibody immunofluorescence was quantified and compared with healthy controls.

**Method:**

Nineteen patients (eight males and eleven females) agreed to be followed-up. Thirteen had the diagnosis of schizophrenia, two had the diagnosis of major depressive disorder, two had bipolar disorder, one had postpartum depression, and one had herpes simplex encephalitis. No patient had catatonia during the follow-up. Nineteen sex-matched healthy controls were recruited.

**Results:**

Using Mann-Whitney U test, patients had greater intensity of anti-NMDAR antibody immunofluorescence than the healthy controls (121,979 ± 86,526 vs. 47,692 ± 26,102, *p* = 0.003). No correlation was found between immunofluorescence intensity and catatonia scales or symptom severity scores. Neuropsychiatric patients with past catatonia showed greater anti-NMDAR antibody response than the healthy controls.

**Conclusion:**

NMDAR dysfunction might play a role in the mechanism underlying catatonia. Further studies are needed to confirm this finding.

## Introduction

Anti- N-methyl-D-aspartate receptor (NMDAR) antibody was thought to be the cause of anti-NMDAR encephalitis [1]. NMDARs are glutamate receptors located in the post-synaptic membrane. Each NMDAR consists of two NR1 (or GluN1) subunits and two NR2/3 subunits. The extracellular epitope of NR1 is crucial for the immune-reactivity [2]. The initial manifestation of anti-NMDAR encephalitis include a wide range of psychiatric symptoms, such as delusions and hallucinations, and those patients were frequently referred to psychiatrists [3]. The acute psychiatric symptoms were then frequently followed by impaired consciousness and alternating catatonia and agitation [3]. In patients with intellectual disability and autism, catatonia and neuroleptic malignant syndrome could be the major manifestations of anti-NMDAR encephalitis [4].

Catatonia is a unique clinical phenomenon characterized by motor, vocal, and behavioral abnormalities such as posturing, negativism, and mutism. Catatonia can be an independent diagnosis [5–7], but it was also associated with a wide range of diagnoses, such as schizophrenia, mood disorders, general medical conditions (GMC) [8], substance withdrawal [9–12], and illicit substances [13]. While catatonia had been frequently described in patients with anti-NMDAR encephalitis, there had been few studies investigating anti-NMDAR antibodies in the patients with catatonia [14]. Anti-NMDAR antibodies were investigated more extensively in patients with schizophrenia, though not all results had been consistent [15–19].

In this study, we aim to investigate anti-NMDAR antibody in neuropsychiatric patients who had catatonia in a follow-up evaluation. The intensity of antibody immunofluorescence was quantified and analyzed.

## Materials and methods

### Subjects and study design

From July 2014 to June 2015, patients who received treatment for catatonia in the past were contacted for a follow-up evaluation. The catatonia of those patients were previously treated with lorazepam and diazepam, and most responded favorably [20–25]. Sex-matched, healthy controls were also recruited. Capacity of consent was determined by ensuring that the individual could understand information about the study, could use that information to decide whether to be recruited or not, and could communicate that decision. Written informed consent was provided by all participants after the content and context of the study was fully explained. The consent procedure was reviewed and approved by the ethics committee. The patients were mostly free of catatonia during the follow-up, but they were evaluated with Bush-Francis Catatonia Rating Scale (BFCRS) and Kanner scales to detect any residual catatonic symptoms. Associated psychiatric diagnosis was determined by two psychiatrists according to the criteria of the Diagnostic and Statistical Manual of Mental Disorders, Fourth Edition (DSM-IV) [26]. The associated diagnoses were categorized into four major groups: schizophrenia, major depressive disorder, bipolar disorder, and organic psychiatric disorder. Post-partum depression was categorized under organic psychiatric disorder because women underwent significant physiological and hormonal turmoil in the post-partum period. The severity of psychiatric disease was evaluated with Positive and Negative Syndrome Scale (PANSS), Hamilton Depression Rating Scale-17 (Ham-D), or Young Mania Rating Scale (YMRS), depending on diagnosis.

Sex-matched healthy controls were screened with the Chinese Health Questionnaire-12 to exclude psychiatric diagnosis [27]. Individuals with a personal or family history (first-degree relatives) of psychiatric disorders and those on psychotropic or anti-inflammatory medication were excluded.

The institutional review board (IRB) of Chang Gung Memorial Hospital approved the study design (IRB 103-1888B). All methods were performed in accordance with the relevant guidelines and regulations.

### Laboratory data

Venous blood (5 ml) was sampled from each participant. Blood samples were placed into regular biochemistry tubes and centrifuged at 4,000x g for 10 minutes. Separated serum samples were then stored at -80°C until the time of the analysis. Indirect immunofluorescence after incubation of serum (1:20 dilution) with human embryonic kidney (HEK293) cells transfected with an expression vector construct containing the recombinant receptor NR1 was used to detect NMDAR antibodies, as described before [28].

### Measurement of immunofluorescence

**Fig 1:**
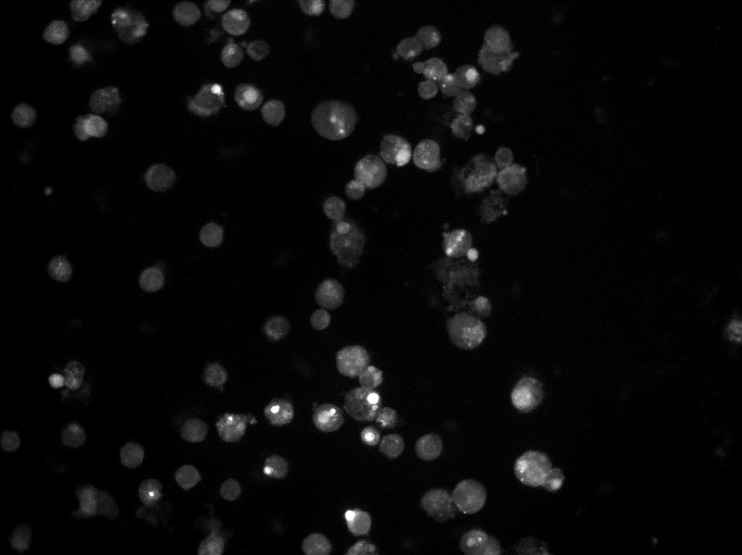
Anti-NMDAR antibody immunofluorescence.

Cells were filmed with a Axiocam MRm camera coupled to a Zeiss AxioImager A2 upright microscope fitted with a Zeiss 40×/0.75 Plan-Neofluar objective and X-Cite 120Q Metal Halide lamp, controlled by Axio Vision SE64 Rel 4.9.1. The resulting images were converted to black and white and analyzed using ImageJ 1.50i (**Fig 1**). Intensity of immunofluorescence was quantified by corrected total cell fluorescence (CTCF), by substracting background signal (average signal per pixel for a region selected just beside the cell) from whole cell signal (sum of the intensity of the pixels for one cell) [29]. For each sample, five CTCFs were measured and the average was calculated for statistical analysis.

### Statistical analysis

All results are represented as mean ± standard deviation. Data analysis was performed using SPSS 19 (Chicago, IL, U.S.A.). The difference of cellular immunofluorescence between patients and controls was compared using Mann-Whitney U test. Pearson correlation was performed to detect correlations between immunofluorescence and clinical parameters. *p* values of <0.05 were considered statistically significant.

## Results

Nineteen patients (eight males and eleven females) agreed to be followed-up. Thirteen had the diagnosis of schizophrenia, two had the diagnosis of major depressive disorder (MDD), two had bipolar disorder, one had postpartum depression, and one had herpes simplex encephalitis. At the time of follow-up, no patient showed prominent catatonic symptoms. One patient had a higher score due to mutism and stereotypic behaviors which could be better explained by negative symptoms. Their data are summarized in **S1 Table**.

Nineteen sex-matched healthy controls were recruited. Their demographic data is shown in **S2 Table**. Using Mann-Whitney U test, the patient group has greater CTCF than the healthy controls (121,979 ± 86,526 vs. 47,692 ± 26,102, *p* = 0.003). No correlation was found between CTCF and age, body mass index (BMI), BFCRS score, Kanner scale score, PANSS, Ham-D, or YMRS (**S3 Table**).

## Discussion

The major finding is that the patient group has greater immunofluorescence than the healthy controls. Whether this difference is attributed to previous catatonia or underlying disease could not be clarified. There had been a few reports regarding catatonia and anti-NMDAR antibody. A recent Japanese study reported two patients with catatonia, and speculated that the presence of anti-NMDAR antibody in CSF could be the cause of catatonic symptoms [14]. Parenti suggested that NMDAR hypofunction in the frontostrial circuitry would cause neural excitory/inhibitory imbalance in the glutamate and GABA pathways could be possible mechanism of catatonia [30]. Inta noted that MK801, a NMDAR antagonist, induced a dose-dependent effects on locomotion, ranging from catatonia to hyperactivity [31]. Those theories require further studies to support, however. Nevertheless, traditional treatment of catatonia, such as benzodiazepines and ECT, was reported to be beneficial in a 14-year-old female with anti-NMDAR encephalitis [32].

A significant portion of our catatonic patients were diagnosed with schizophrenia. The relationship between schizophrenia and ant-NMDAR antibody had been investigated more extensively in the past, though the results have not been entirely consistent. While some earlier reports found no anti-NMDAR antibody in patients with schizophrenia [16, 33, 34], others reported otherwise [15, 35]. A study including 121 patients with schizophrenia, 70 MDD, 38 borderline personality disorder (BLPD), and 230 controls found diverse anti- NMDAR antibodies in 15 subjects, primarily those with an initial schizophrenia diagnosis (9.9%), as opposed to MDD (2.8%), BLPD (0), and controls (0.4%) [19]. A systematic, quantitative review of those six studies found a significantly increased prevalence of serum anti-NMDAR antibodies in patients with schizophrenia [36].

In a study consisting of 2817 subjects (1325 healthy, 1081 schizophrenic, 263 Parkinson, and 148 affective-disorder subjects), anti-NMDAR antibodies were detected using commercial biochips in sera of 10.5% of the study subjects, with no statistically significant difference in prevalence between patients and healthy controls [37]. Steiner re-tested his 2013 sample with the commercial biochips, and found increased prevalence of anti-NMDAR antibody in all groups, especially in the healthy controls, which rendered the difference between groups insignificant [17]. An explanation of this increased seroprevalence was that cell-based assays using fixed cells (the commercial biochips) appear to be more sensitive than cell-based assays using live transfected human embryonic kidney cells [38]. Fixed cells no longer retain intact cell membranes, thus the detected anti-NMDAR antibodies could target regions other than the cell surface epitopes of the NMDAR [39]. A meta-analysis found that prevalence rates were greater in patients with schizophrenia and related psychoses than controls only for IgG antibodies, and also noted the heterogeneity between cell-based assays utilized by different research groups [40]. Another meta-analysis suggested that higher dilution rates were needed to differentiate anti-NMDAR antibody seropositivity between patients with schizophrenia, schizoaffective disorder, bipolar disorder, or major depressive disorder from healthy controls [18]. However, those meta-analyses did not use the updated data from Steiner's re-test, so the results might be skewed [39].

In a study of 1703 healthy controls and 2533 patients (of schizophrenia, affective disorders, stroke, Parkinson's disease, amyotrophic lateral sclerosis, and personality disorder) using the commercial biochips found similar serum anti-NMDAR antibody prevalences (about 10%) between patients and controls [41]. Using the similar commercial biochips, a study including 50 patients with first episode psychosis and 50 controls found no anti-NMDAR antibody in the serum of any participant [42]. No anti-NMDAR NR1 antibody was detected in the CSF of 49 patients with schizophrenia and 48 healthy controls, using the commercial biochips with either 1/10 (24 patients and 24 controls) and 1/200 (25 patients and 24 controls) dilution rates [43]. In a study of patients with narcolepsy type 1 (NT1), no IgG antibodies to NR1/NR2B heteromers of the NMDARs was detected in patients with NT1 with or without psychosis [44]. Another recent study in Taiwan found no positive anti-NMDAR autoantibody in plasma of patients with schizophrenia [45]. Taken together, the role of anti-NMDAR antibody in schizophrenia remains unclear.

The pathogenicity of antibodies have been investigated. Human CSF-derived monoclonal NR1 antibodies alone could cause receptor downregulation and subsequent impairment of NMDAR-mediated currents on primary [46]. Serum with NMDAR antibody of any immunoglobulin class could also provoke NMDAR internalization and reduction of glutamate evoked currents in NMDAR-expressing Xenopus oocytes [47]. In that case, the unexpectedly high prevalence of serum anti-NMDAR antibody in healthy controls in some studies were puzzling [17, 37, 41]. Blood-brain barrier (BBB) integrity might prevent serum autoantibody from affecting the CNS [37]. In a study monitoring both apolipoprotein E4 (an indicator of preexisting leaky BBB) and anti-NMDAR antibody in patients with acute ischemic brain injury, the outcomes were different according to BBB integrity [48]. Translational study on mice also showed that brain could behave as an immunoprecipitator of anti-NMDAR antibodies [49].

There had been no standardized examination to detect anti-NMDAR antibody. Cell-based assays employed by different research groups differ in dilution rates, cell status (live or fixed), etc[40]. In earlier publications, the detection of anti-NMDAR antibodies required transfection of NMDAR cDNA into HEK293 cells, incubate the resulting cells with serum or CSF, then indirectly detect the attached antibody [28, 50]. The commercial biochip simplifies the steps, and have been utilized increasingly in the recent years. The commercial biochip also has the advantages of identifying different classes of antibodies as well as detecting the antibody titers with serial dilution. The commercial biochip would detect antibodies targeting non-surface region of the NMDAR, however [39]. The main disadvantage of the commercial biochip might be its price tag, which could be unaffordable for less well-funded laboratories. Two recent studies employed double-labeling with a commercial anti-NMDAR NR1 antibody [14, 46], which would be an interesting alternative. In this study, we continued to use the traditional, live cell-based assay, but tried to quantify the intensity of immunofluorescence with the freely available software ImageJ [29]. The anti-NMDAR antibodies in study subjects were described as either positive or negative [28, 50], though some papers described them with titers [19], offering more freedom in analysis. Our method attempted to directly quantify the intensity of immunofluorescence of anti-NMDAR antibody, offering more direct comparison.

There are several limitations of this study. The sample size is small. The age-matched control group is younger than the patient group. An earlier study had suggested that seroprevalence of anti NMDAR antibodies is age-related [51]. The study sample was heterogenous, composed of various associated diagnoses, and the mechanism of developing catatonia in schizophrenia and in mood disorder could be quite different. Patients willing to be followed-up for catatonia re-evaluation were those still undergoing treatment in our hospital, mostly because of schizophrenia, thus the difference of immunofluorescence intensity could be caused by the underlying diseases. The study design was retrospective, and the measurement of anti-NMDAR antibody took place years after the last catatonia, so they might not be directly related. None of the patients were treated with treatments specifically targeting anti-NMDAR antibody (such as IVIG and plasmapheresis) during their catatonia, as a positive response to those immunotherapies could support that catatonia and anti-NMDAR antibody are directly connected. The inclusion of a patient of Herpes Simplex Encephalitis was due to the rarity of catatonic patients in the past few decades, so we included as many catatonic patients as possible despite the heterogeneity. Lastly, the relationship between anti-NMDAR antibody CTCF and antibody titer remains unclear, though a cut-off point for CTCF would be interesting, the analysis was not performed due to small sample size.

## Conclusion

Prolonged catatonia could lead to complications such as malnutrition and rhabdomyolysis. In catatonic patients with rapidly deteriorating physical conditions, anti-NMDAR encephalitis should be suspected, and the confirmation of diagnosis by detecting anti-NMDAR autoantibody would be beneficial. Our data suggested that NMDAR dysfunction might play a role in the mechanism underlying catatonia. Further studies are needed to confirm this finding.

## Supporting information

S1 Table Patients with past catatonia. BFCRS: Bush-Francis Catatonia Rating Scale; CTCF: corrected total cell fluorescence; MDD: major depressive disorder.
(DOC)

S2 Table Demographic data of patients and healthy controls. BMI: body mass index; CTCF: corrected total cell fluorescence.
(DOC)

S3 Table Correlations between variables. In each cell Pearson's r and *p* value were shown in the first and second row, respectively. BFCRS: Bush-Francis Catatonia Rating Scale; BMI: body mass index; CTCF: corrected total cell fluorescence; Ham-D: Hamilton Depression Rating Scale-17; YMRS: Young Mania Rating Scale.
(DOC)
