# Supplementary material for: Increased serum anti-N-methyl-D-aspartate receptor antibody immunofluorescence in psychiatric patients with past catatonia
Source: PLoS One. 2017 Oct 26;12(10):e0187156. doi: 10.1371/journal.pone.0187156 (PMC5658162; doi:10.1371/journal.pone.0187156)
Supplement: S1 Table — BFCRS: Bush-Francis Catatonia Rating Scale; CTCF: corrected total cell fluorescence; MDD: major depressive disorder. (DOC) [file pone.0187156.s001.doc]

S1 Table. Patients with Past Catatonia

| Associated Diagnosis | Age | Sex | Onset of Associated Diagnosis | BFCRS Score | KANNER Score | Age of First Catatonia | Age of Last Catatonia | Average CTCF |
| --- | --- | --- | --- | --- | --- | --- | --- | --- |
| Schizophrenia (n = 13) |  |  |  |  |  |  |  |  |
|  | 27 | F | 18 | 4 | 4 | 24 | 24 | 194292 |
|  | 32 | F | 20 | 11 | 18 | 22 | 22 | 68026 |
|  | 36 | F | 27 | 5 | 4 | 27 | 30 | 106219 |
|  | 36 | M | 29 | 0 | 0 | 31 | 31 | 232421 |
|  | 40 | M | 21 | 0 | 0 | 29 | 32 | 152533 |
|  | 40 | F | 28 | 0 | 0 | 39 | 39 | 272224 |
|  | 45 | M | 20 | 2 | 4 | 39 | 39 | 173217 |
|  | 47 | F | 18 | 3 | 0 | 33 | 33 | 180079 |
|  | 48 | F | 30 | 0 | 0 | 33 | 40 | 153995 |
|  | 48 | F | 33 | 0 | 0 | 33 | 33 | 10625 |
|  | 51 | F | 30 | 3 | 0 | 44 | 44 | 93162 |
|  | 57 | M | 38 | 1 | 0 | 43 | 45 | 111720 |
|  | 58 | M | 43 | 0 | 0 | 46 | 46 | 36087 |
| MDD (n = 2) |  |  |  |  |  |  |  |  |
|  | 45 | F | 37 | 5 | 0 | 40 | 44 | 695 |
|  | 57 | M | 36 | 1 | 0 | 46 | 46 | 51954 |
| Bipolar (n = 2) |  |  |  |  |  |  |  |  |
|  | 51 | M | 25 | 0 | 0 | 47 | 47 | 58672 |
|  | 66 | M | 54 | 3 | 0 | 61 | 61 | 87378 |
| Organic (n = 2) |  |  |  |  |  |  |  |  |
| Postpartum | 38 | F | 25 | 0 | 0 | 25 | 30 | 36716 |
| Herpes simplex encephalitis | 51 | F | 37 | 0 | 0 | 37 | 50 | 297593 |

BFCRS: Bush-Francis Catatonia Rating Scale; CTCF: corrected total cell fluorescence; MDD: major depressive disorder
