# Supplementary material for: Increased serum anti-N-methyl-D-aspartate receptor antibody immunofluorescence in psychiatric patients with past catatonia
Source: PLoS One. 2017 Oct 26;12(10):e0187156. doi: 10.1371/journal.pone.0187156 (PMC5658162; doi:10.1371/journal.pone.0187156)
Supplement: S2 Table — BMI: body mass index; CTCF: corrected total cell fluorescence. (DOC) [file pone.0187156.s002.doc]

S2 Table. Demographic data of patients and healthy controls

|  | Age | BMI | CTCF |
| --- | --- | --- | --- |
| Patients  (n = 19) | 46.0 ± 9.9 | 25.8 ± 3.9 | 121,979 ± 86,526 |
| Controls  (n = 19) | 32.3 ± 5.3 | 22.2 ± 3.4 | 47,692 ± 26,102 |
|  |  |  | *p* = 0.003* |

BMI: body mass index; CTCF: corrected total cell fluorescence
