# Supplementary material for: Increased serum anti-N-methyl-D-aspartate receptor antibody immunofluorescence in psychiatric patients with past catatonia
Source: PLoS One. 2017 Oct 26;12(10):e0187156. doi: 10.1371/journal.pone.0187156 (PMC5658162; doi:10.1371/journal.pone.0187156)
Supplement: S3 Table — In each cell Pearson's r and p value were shown in the first and second row, respectively. BFCRS: Bush-Francis Catatonia Rating Scale; BMI: body mass index; CTCF: corrected total cell fluorescence; Ham-D: Hamilton Depression Rating Scale-17; YMRS: Young Mania Rating Scale. (DOC) [file pone.0187156.s003.doc]

**S3 Table.** Correlations between variables

|  | (1) | (2) | (3) | (4) |
| --- | --- | --- | --- | --- |
| (1) CTCF |  |  |  |  |
| (2) Age | 0.157  0.346 |  |  |  |
| (3) BMI | 0.212  0.202 | 0.275  0.095 |  |  |
| (4) BFCRS | -0.223  0.358 | -0.367  0.122 | 0.183  0.454 |  |
| (5) Kanner | -0.057  0.823 | -0.499  **0.033** | 0.128  0.600 | 0.842  **0.000** |
| (6) PANSS | 0.084  0.794 | -0.671  **0.017** | 0.055  0.866 | 0.620  **0.032** |
| (7) Ham-D | -0.496  0.504 | 0.071  0.929 | 0.180  0.820 | 0.458  0.542 |
| (8) YMRS | -0.077  0.951 | -0.041  0.974 | 0.403  0.736 | -0.500  0.667 |

In each cell Pearson's r and *p* value were shown in the first and second row, respectively.

BFCRS: Bush-Francis Catatonia Rating Scale; BMI: body mass index; CTCF: corrected total cell fluorescence; Ham-D: Hamilton Depression Rating Scale-17; YMRS: Young Mania Rating Scale
